# Barriers and facilitators of access and utilization of mental health services among forensic service users along the care pathway

**DOI:** 10.1186/s12913-022-08848-9

**Published:** 2022-12-07

**Authors:** Marichelle C. Leclair, Yanick Charette, Michael Seto, Tonia L. Nicholls, Laurence Roy, Mathieu Dufour, Anne G. Crocker

**Affiliations:** 1 Department of Psychology & School of Public Health, Université de Montréal, Montréal, Canada; 2 Institut national de psychiatrie légale Philippe-Pinel, Montréal, Canada; 3 School of Social Work and Criminology, Université Laval, Québec City, Canada; 4 Forensic Research Unit, Royal Ottawa Health Care Group, Ottawa, Canada; 5 Department of Psychiatry, University of British Columbia & British Columbia (BC) Mental Health & Substance Use Services, Vancouver, Canada; 6 School of Occupational Therapy, McGill University, Montréal, Canada; 7 Department of Psychiatry and Addictology & School of Criminology, Université de Montréal, Montréal, Canada

**Keywords:** Access to care, Engagement, Family-centred care, Mental health, Stigma

## Abstract

**Background:**

The verdict of Not Criminally Responsible on account of a Mental Disorder (NCRMD) is increasingly used to access specialized mental health services in Canada and elsewhere. This situation highlights the importance of ensuring timely access to services in the community to prevent violence and justice involvement. The objective of the present study is to identify individual and contextual barriers and facilitators of access to mental health services during the period preceding an offense leading to a verdict of NCRMD.

**Methods:**

The sample includes 753 people found NCRMD in Québec, Canada. All episodes of mental health hospitalizations and service use before the index offense were identified using provincial administrative health data, for an average period of 4.5 years. Access was conceptualized as a function of the possibility of seeking, reaching and receiving appropriate health care services, based on Lévesque and colleagues patient-centred model of access to care.

Generalized linear models were computed to identify the individual and contextual predictors of: (1) *seeking* mental healthcare (at least one contact with any type of services for mental health reasons); (2) *reaching* psychiatric care (at least one contact with a psychiatrist); (3) *receiving* psychiatric care, operationalized as (3a) continuity and (3b) intensity. Factors associated with volume of emergency mental health services were examined as exploratory analysis.

**Results:**

Geographical considerations were highly important in determining who reached, and who received specialized mental health care – above and beyond individual factors related to need. Those who lived outside of major urban centres were 2.6 times as likely to reach psychiatric services as those who lived in major urban centres, and made greater use of emergency mental health services by 2.1 times. Living with family decreased the odds of seeking mental healthcare by half and the intensity of psychiatric care received, even when adjusting for level of need.

**Conclusions:**

Findings support efforts to engage with the family of service users and highlights the importance of providing resources to make family-centred services sustainable for health practitioners. Health policies should also focus on the implementation of outreach programs, such as Forensic Assertive Community Treatment teams as part of prevention initiatives.

**Supplementary Information:**

The online version contains supplementary material available at 10.1186/s12913-022-08848-9.

The criminal justice system has been increasingly used to access mental health services in a timely manner in Canada and elsewhere [1–3], as illustrated by the remarkable growth in the number of verdicts of non criminal responsibility on account of a mental disorder (NCRMD) and associated admissions to forensic mental health services [4, 5]. In Canada, a person is found NCRMD when their psychiatric symptoms made it so that they were “incapable of appreciating the nature and quality of the act [...] or of knowing that it was wrong” [6]. In the province of Québec, the verdict has been used more extensively than in other provinces as a lever to access specialized mental healthcare. Indeed, the NCRMD defense is used in Québec for offenses of lesser severity and with persons with a greater diversity of diagnoses [7]. This *forensication* of mental health services [8] results in important implications for the persons and their loved ones, including additional stigma [9] and greater privation of liberty [10]. In addition, treating a patient in forensic mental health services incurs costs five times greater compared to general mental health services [11].

Better access to responsive, integrated and equitable mental health services has been highlighted time and time again as a priority for provincial Canadian healthcare systems [12–14], especially for people who are considered at high risk of committing an offense due to their mental illness symptoms [15]. The situation related to the COVID-19 pandemic has exacerbated these issues, accelerating the fragilization of certain groups and of health systems, resulting in a deterioration in accessibility of mental health services [16, 17]. People who have a severe mental illness and who are at risk of committing an offense are likely to experience several of the barriers to mental health services identified in the literature [18] in ways that pose unique challenges [19]. For example, those service users may have concerns about stigma or experience discrimination within services [20–23] as they are labeled as “dangerous” or “too difficult” by providers [24]. They often have a history of criminal justice involvement [7] and several concurrent diagnoses [25] which, in addition to lack knowledge of available resources [26], may make fragmented services and complex care pathways [27] even more difficult to navigate.

This situation highlights the importance of ensuring access to mental health services in the community and of understanding the barriers and facilitators along pathways to care for these high-need service users. Lévesque et al.’s patient-centered model of access to health care [28], and the older Goldberg and Huxley model [29], have emphasized the importance of operationalizing access to care in multilevel ways that encompass the possibility of recognizing healthcare needs, seeking services, reaching healthcare resources, and receiving services that are relevant and appropriate to the individual’s healthcare needs, where relevance and appropriateness can be understood through the lense of quality (e.g., continuity) or adequate intensity [28]. This allows an account of the entire experience of service users across the healthcare system. Different models of access to care [28, 30], including Andersen’s behavioral model of health services use [31–33], have emphasized that both service-level (e.g., geographic location of services, availability of services, referral mechanisms, coordination of care) and individual-level (e.g., social support, criminal history, housing) variables are relevant to informing our understanding of how and by whom health care services are accessed and used, above and beyond health needs.

The objective of the present study is to identify individual and contextual barriers and facilitators of access to mental health services specifically during the period preceding an offense leading to an NCRMD verdict (index offense). For the purpose of the present investigation, we borrow from Lévesque and colleagues’ definition and conceptualisation of access. We define access as the “opportunity to have healthcare needs fulfilled” [28] as a function of the possibility of *seeking*, *reaching* and *receiving* appropriate healthcare services.

## Methods

### Sample and procedures

Data were extracted from the Québec sample of the National Trajectory Project [34], a longitudinal file-based study of 1094 people declared NCRMD between 2000 and 2005. Given our focus on the period prior to the index offense and on community healthcare service use, we excluded service users for whom matching with administrative health services data was unsuccessful (*n* = 74), for whom the exact date of the index offense was unknown (*n* = 6), who were in an institution at the time of the index offense (*n* = 53) or homeless (*n* = 86), who lived outside of the province of Québec (*n* = 5), or for whom the address at the time of the offense was missing (*n* = 106). Address matching with data from *Institut national de santé publique du Québec* (Québec Public Health Institute, INSPQ) [35] (which provided data regarding proximity to services, for example – see *Measures* below) was successful for 753 of the eligible participants. The present analyses therefore involve 753 service users who were housed in the community at the time of their index offense. The majority of participants were men (85%), 84% were single, only 17% had employment or a partner’s employment as main source of revenue, and 45% had prior criminal history (see Table 1). In terms of clinical characteristics, at the verdict, 64% had a primary diagnosis of psychotic disorder, 30% of mood disorder (most frequently bipolar disorder), and 6% had another primary diagnosis. Concurrent personality disorder was found in 9.7% of service users, and concurrent substance use disorder in 30.0%. There were no notable differences between included and excluded participants (see Table S1 of the online supplement for a detailed comparison).

**Table 1:** Characteristics and index offenses of participants

Variables	Participants (*n* = 753)
Gender, %	
Women	14.5
Men	85.5
Age, *M* (*SD*)	36.7 (12.4)
Civil status, %	
In a relationship	15.7
Single	84.3
Born in Canada, %	62.7
Indigenous, %	1.0
Revenue, %	
Own paid work (or partner’s)	16.7
Pensions, welfare, disability	74.8
Other	8.5
Primary diagnosis, %	
Psychotic disorder	64.2
Mood disorder	29.6
Other	6.2
Concurrent personality disorder, %	9.7
Concurrent substance use disorder, %	30.0
Most severe index offense, %	
Causing death or attempting to cause death	4.9
Sexual offense	1.5
1st degree assault	5.2
Other assault	19.8
Threats	22.0
Other against the person	8.2
Property offenses	19.9
Administration of justice	4.9
Other	13.7
Criminal history, %	45.0
NCRMD history, %	8.5

Weighted statistics

The research protocol was approved by appropriate institutional ethics review committees. Governmental health records were received through the Québec Access to Information Commission (*Commission d’accès à l’information*).

### Measures

Administrative health services data for the 5 years prior to the NCRMD verdict were received from the Ministry of Health and Social Services’ MED-ECHO and the provincial medical insurance plan (*Régie de l’assurance maladie du Québec*; RAMQ), a physician fee-for-service database on all medical interventions completed under the public insurance system. We selected all hospitalizations and services before the index offense, up to the day before, for an average observation period of 4.53 years (*SD* = 0.55, minimum = 0.77 years, maximum = 5.0 years – less than 1% of the sample had a follow-up time shorter than 2 years and 84.8% had a follow-up time longer than 4 years). Given that provinces in Canada rely on universal, public, single-payer systems, these data are available for medical services received in Québec in the public system. Given that only 15.7% of our sample had paid employment and that there are very few psychiatrists whose services are not covered by the RAMQ [36], we estimate that the proportion of participants who may have used private healthcare – which is not covered by private or public insurance – to meet their health needs is minimal. Hospitalisation associated with a primary or secondary diagnosis of mental disorder (ICD-9 codes 290–319, which includes substance use disorders) and services associated with a primary diagnosis of mental disorder were classified as “mental health related”. Consistent with the literature [37], multiple medical services provided for the same diagnosis, on the same day and at the same institution or by the same provider were coded as a single visit.

Three categories of data were coded from the Review Board^1^ files: (1) sociodemographic data (e.g., age, gender, people with whom they lived at the time of the index offense, address of residence at the time of the index offense); (2) clinical data (e.g., diagnoses at the NCRMD verdict); and (3) judicial history (e.g., nature and date of index offenses, presence of past NCRMD offenses). Criminal history was identified from the files of the Royal Canadian Mounted Police criminal records (for a full explanation, see Crocker et al., 2015 [34]).

#### Dependent variables

Outcome variables represent different levels of access to care. First, the concept of “*seeking* mental healthcare” was operationalized as a dichotomous variable where those who had at least one contact with any type of services for mental health reasons prior to their index offense were considered to have “successfully” sought care. This operationalization relies on the assumption that all people in our sample had a need for mental healthcare, which is a reasonable assumption given that all people found NCRMD must have had a mental disorder that rendered them unable to appreciate the nature and quality of the act or of knowing that it was wrong, and that 72.5% of people found NCRMD in Québec had been hospitalized for a mental health problem in their lifetime [7]. However, there may be a small proportion of people for whom the index offense was the first manifestation of their mental illness [38].

Second, we operationalized “*reaching* psychiatric care” as having had at least one contact with a psychiatrist, on an outpatient or inpatient basis, prior to the index offense. Given the vast majority of people with a NCRMD verdict had a severe mental illness (i.e., psychotic spectrum disorder or bipolar disorder; see Table 1), this operationalization assumes that all those who sought contact with the healthcare system for a mental health reason should have been referred to a psychiatrist. As in most provinces, psychiatric care in Québec is accessible almost solely through referral from a primary care physician or through psychiatric emergency services.

Third, we operationalized “*receiving* psychiatric care” as two distinct outcomes used as proxy for intensity and quality of services: volume of psychiatric care (sum of psychiatric visits [consultations and follow-up], as an inpatient or outpatient) and continuity of psychiatric care. We calculated the Bice-Boxerman continuity of care index [39], which reflects the extent to which regular psychiatric care is provided by a single psychiatrist, outside of hospitalization periods and emergency room visits. This index takes continuous values from 0 to 1; if all psychiatric visits were exclusively with the same provider, the index would be 1, whereas all visits to different providers would result in an index of 0. To calculate continuity, we used only evaluation and management visits, as suggested in the literature [39].

As an exploratory analysis, we sought to identify contextual and individual factors related to volume of emergency mental health services among those who had had at least one contact with services for mental health reasons (sum of emergency room visits where a service was provided for mental health reasons). The reason we decided to frame it as an exploratory analysis rather than an indicator of *receiving* psychiatric care is that emergency room visits can both be interpreted as an indicator of access in times of distress and crisis or as an indicator of lack of access (for example, if a person is unable to access services in a timely manner until the situation requires emergency care, or if a person seeks routine mental healthcare through the emergency room).

#### Independent variables

Contextual and individual independent variables were selected based on the factors put forward by the behavioral model of health services use [32].

For contextual factors, based on the address of the participant at the time of the verdict, we considered the annual expenditure in health and social services per capita, the number of physicians per 1000 population in every regional health district [40], whether the area of residence was considered as part of a major urban centre or not, as per the INSPQ, and proximity to services (measured using the number of physician offices and local community service center within a 15 minute drive radius, the number of hospital centers providing outreach services within a 30 minute drive radius, or the number of hospital centers providing services for complex conditions upon referral within a 60 minute radius, as calculated by the INSPQ [35]). Outreach services aim to improve the availability of services and the coordination of care through initiatives such as community health workers or mobile clinics [41]. We also considered the index of social and material deprivation of the area of residency, which are represented using quintiles of deprivation [42].

For individual factors, we considered the following predisposing and enabling factors: age at the start of the observation period, gender, if the person lived with relatives at the time of the index offense (i.e., a partner, a family members or friends, as proxy for social support), having a criminal record, having a significant connection to a primary care physician (understood as a Usual Provider Care index [43] greater than 75% or a complete medical examination by the same provider at least once every 2 years, consistent with proposed algorithms [44]). Factors related to mental health need included the presence of a history of a prior NCRMD verdict, and the diagnoses by the psychiatrist at verdict.

### Analytical plan

We conducted different multivariate generalized linear models for each outcome, adjusting for the length of the observation period as exposure variable. To limit the bias in the association of distal correlates due to the inclusion of intermediates in a single model [45], we opted for a series of regressions entering blocks of variables one at a time, progressing from the most distal covariates to the most proximal covariates (block 1: contextual factors; block 2: individual predisposing and enabling factors; block 3: individual need-related factors). A similar strategy has been used in other studies examining predictors of access to mental health services [46, 47]. We used the binomial function for *seeking* and *reaching*, the gamma function for continuity of care and the negative binomial function for volume of psychiatric care and volume of emergency mental health services. All analyses used sample weights.

#### Sensitivity analysis

To account for the fact that the intensity of psychiatric care received (i.e., volume of psychiatric care) may be both indicative of greater access and of greater mental health need, we adjusted for the annual number of days in psychiatric hospitalization as a proxy for mental health needs. The results of this sensitivity analysis are reported in text.

## Results

Half of participants lived in a major urban centre (50.5%) at the time of the index offense, and a disproportionate proportion lived in neighborhoods of the most materially deprived (34.5%) or socially deprived (34.2%) quintile. On average, they were hospitalized for psychiatric reason 8.2 days every year (*SD* = 18.2), and only 18.0% were considered as having a significant connection to a family physician based on the algorithm.

### Seeking mental healthcare

Of the full sample *(n* = 753), as many as 87.0% of participants sought mental healthcare in the average 4.5 year observation period prior to their index offense. No contextual characteristics increased the odds of seeking care, but individual factors did (see Table 2). Among predisposing factors, presence of a criminal history (OR = 2.20, *p* = .004) and having an connection to a general practitioner (OR = 3.58, *p* = .001) increased the odds of seeking care for mental health reasons, whereas living with family or a partner was associated with a reduction in healthcare seeking (OR = 0.53, *p* = .012). No factors associated to need predicted seeking care.

**Table 2:** Barriers and facilitators to mental healthcare access in the 4.5 years before a NCRMD offense

Variables	*Seeking* mental health care (*N* = 753)		*Reaching* psychiatric care (*N* = 661)		*Receiving*: Volume of psychiatric care (*N* = 555)		*Receiving*: Continuity of psychiatric care (*N* = 437)		Volume of emergency mental healthcare (*N* = 661)	
	OR	95% CI	OR	95% CI	RR	95% CI	*β*	95% CI	RR	95% CI
Block 1: Contextual										
Physicians per 1000 pop.	1.16	0.64, 2.09	**1.84**	**1.15, 2.85**	**1.31**	**1.02, 1.67**	0.07	−0.14, 0.27	1.20	0.91, 1.59
Residency outside of major urban centres	1.36	0.55, 3.35	**2.61**	**1.20, 5.66**	0.78	0.52, 1.19	0.13	−0.25, 0.52	**2.06**	**1.40, 3.02**
Material disadvantage of residency area	0.84	0.70, 1.00	0.93	0.78, 1.10	0.95	0.87, 1.04	−0.00	−0.08, 0.07	0.91	0.83, 1.00
Social disadvantage of residency area	1.12	0.95, 1.34	0.92	0.78, 1.08	1.03	0.95, 1.12	0.07	−0.01, 0.13	0.98	0.87, 1.11
Community ressources within 15 min.	1.00	1.00, 1.00	1.00	1.00, 1.00	1.00	1.00, 1.00	−0.00	−0.00, 0.00	1.00	1.00, 1.00
Hospital centres offering outreach services within 30 min.	1.00	0.74, 1.35	1.31	0.99, 1.71	**0.86**	**0.75, 0.98**	**0.18**	**0.01, 0.36**	1.01	0.87, 1.17
Hospital centres offering referral services within 60 min.	1.00	0.92, 1.08	**1.11**	**1.04, 1.18**	0.99	0.96, 1.02	−0.02	−0.05, 0.01	1.02	0.99, 1.06
Block 2: Predisposing										
Age	0.99	0.97, 1.02	**0.98**	**0.96, 1.00**	0.99	0.98, 1.00	**−0.01**	**−0.02, − 0.00**	**0.98**	**0.98, 0.99**
Female gender	1.56	0.65, 3.71	1.39	0.77, 2.53	1.10	0.77, 1.56	0.04	−0.23, 0.32	**1.67**	**1.13, 2.48**
Living with a partner, family or friends	**0.53**	**0.33, 0.87**	0.64	0.83, 1.06	**0.73**	**0.59, 0.91**	0.04	−0.17, 0.25	0.89	0.69, 1.16
Criminal history	**2.20**	**1.29, 3.75**	1.15	0.73, 1.81	1.06	0.86, 1.31	−0.07	−0.27, 0.11	**1.29**	**1.01, 1.64**
General practitioner connection	**3.58**	**1.75, 7.36**	0.87	0.52, 1.45	0.82	0.65, 1.04	0.19	−0.06, 0.43	0.95	0.71, 1.26
Block 3: Need										
NCR history	2.30	0.65, 8.13	**16.3**	**2.16, 123.0**	**1.86**	**1.43, 2.41**	0.13	−0.15, 0.40	**1.77**	**1.08, 2.90**
Psychotic disorder	1.56	0.66, 3.71	**2.29**	**1.12, 4.68**	**1.71**	**1.07, 2.75**	0.21	−0.19, 0.62	0.71	0.44, 1.14
Mood disorder	1.45	0.57, 3.65	1.86	0.88, 3.97	1.45	0.88, 2.38	0.06	−0.35, 0.48	0.65	0.41, 1.06
Other disorders	1.32	0.59, 2.96	0.70	0.35, 1.39	1.24	0.87, 1.77	0.26	−0.26, 0.79	0.77	0.54, 1.08
Concurrent personality disorder	0.76	0.35, 1.65	2.24	0.87, 5.71	1.02	0.77, 1.36	−0.13	−0.41, 0.15	1.31	0.95, 1.81
Concurrent substance use disorder	1.39	0.81, 2.37	2.53	1.39, 4.60	0.85	0.68, 1.07	**0.26**	**0.00, 0.52**	**1.31**	**1.04, 1.66**

Exact *p*-values of statistically significant variables (bolded) are provided in text. Seeking care: At least one contact with any medical services for mental health reasons. Reaching psychiatric care: At least one contact with a psychiatrist. The model for seeking care used the full sample (*n* = 753), whereas the models for reaching care and volume of emergency care included those who had sought care (*n* = 661). The model for receiving – volume of psychiatric care included all those who had reached psychiatric care (*n* = 555) whereas the model for receiving – continuity of psychiatric care included only those with at least two psychiatric visits (*n* = 437)

### Reaching psychiatric care

Among the participants who sought services for mental health reasons (*n* = 661), 85.9% reached psychiatric care (i.e., at least one contact with a psychiatrist). Several contextual factors increased the odds of reaching psychiatric care: the number of physicians per 1000 residents of the area of residence (OR = 1.84, *p* = .010), living outside of a major urban centre (OR = 2.61, *p* = .016), and proximity to hospitals providing outreach (OR = 1.31, *p* = .055) or referral services (OR = 1.11, *p* = .003). Living with family or a partner marginally reduced the odds of reaching psychiatric care (OR = 0.64, *p* = .081). Finally, several factors related to mental health needs were predictive of reaching psychiatric care: having a history of forensic involvement (OR = 16.3, *p* = .007), a diagnosis of psychotic spectrum disorder (OR = 2.29, *p* = .024) or of concurrent substance use disorder at verdict (OR = 2.53, *p* = .002). Of note, availability of physicians, living outside of a major urban centre, and proximity to services continued to have an effect above and beyond those need-related factors, with very stable size effects.

### Receiving psychiatric care

Participants who had reached psychiatric services (*n* = 555) consulted on average annually a psychiatrist 9.1 times (*SD* = 11.6) in any context. Among contextual factors, living in an area with a greater number of physicians was associated with more visits to a psychiatrist (RR = 1.31, *p* = .033), whereas greater proximity to hospitals providing outreach services was associated with fewer visits (RR = 0.86, *p* = .026). In terms of individual factors, living with family or friends reduced the volume of psychiatric consults (RR = 0.73, *p* = .006) whereas a NCRMD history (RR = 1.86, *p* < .001) and a diagnosis of psychotic disorder at verdict (RR = 1.71, *p* = .026) increased it. Effect sizes related to proximity to services remained stable when inserting variables related to predisposition or need. As sensitivity analysis, we adjusted for days in psychiatric hospitalization as a proxy for need, to ensure that the factors aforementioned were related to access rather than than need. Adding this variable to the model resulted in two changes: material disadvantage was associated with fewer psychiatric visits (OR = 0.92, *p* = .042) and psychotic disorder was no longer a significant variable.

Of the 437 participants who had at least two contacts with a psychiatrist, the Bice-Boxerman index indicating continuity of psychiatric care (outside of hospitalization periods and emergency room visits) was 0.62 (*SD* = 0.37) for an average of 2.0 different psychiatrists (*SD* = 1.44). Three factors were associated with continuity of psychiatric care. Proximity to outreach services (*β* = 0.18, *p* = .046) and concurrent substance use disorder (*β* = 0.26, *p* = .047) increased continuity of care, whereas age decreased it (*β* = − 0.01, *p* = .011). The effect of proximity to outreach services remained significant with a stable effect size when adding individual factors.

### Exploratory analysis: volume of emergency mental health services

The model for volume of emergency mental healthcare used as a target sample all those who had sought mental health services (*n* = 661). Annually, on average, participants visited an emergency room 1.3 times (*SD* = 2.15) for mental health reasons. Living outside of major urban centres was a predictor of greater use of emergency mental health services (RR = 2.05, *p* < .001), whereas greater social deprivation of the area of residency marginally decreased the use (RR = 0.91, *p* = .052). Age (RR = 0.98, *p* = .002), female gender (RR = 1.67, *p* = .011), a criminal history (RR = 1.29, *p* = .043), a prior NCRMD finding (RR = 1.77, *p* = .022) and concurrent substance use disorder (RR = 1.31, *p* = .024) were also associated with volume of emergency mental health services in the observation period.

When adding greater continuity of psychiatric care (outside of periods of hospitalization or emergency room visits) in the predisposing factors block, thus limiting the model to the 436 service users with at least two visits to a psychiatrist, it was found that greater continuity of care was associated with reduced use of emergency mental health services (RR = 0.64, *p* = .020).

## Discussion

This paper provides an overview of who accesses and receives various types of mental health medical services, in a sample of participants who were selected as a result of being found NCRMD. Traditional models of access to care highlight that needs are the main predictors of service utilization [48]; however, that was not the case here. While facilitators and barriers may differ for every level of care, general trends emerged: the person’s living situation, both in terms of geography and in terms of people with whom the person lived, had major influences on what services were accessed and used or not, even when adjusting for need-related variables such as primary and concurrent diagnoses. There was one major exception in terms of need-related variable: a history of NCRMD was by far the largest size effect in determining who reached psychiatric services, and was a significant predictor of volume of psychiatric care and of emergency mental healthcare. These findings show that a NCRMD verdict changes how service users interact with the mental health system.

Geographical considerations were highly important in determining who reached, and who received, psychiatric care – even when including individual factors related to need in the models. All else – including proximity to services – being equal, those who lived outside of major urban centres were 3 times as likely to reach psychiatric services as those who lived in major urban centres. They were also 2 times more likely to frequently visit the emergency room for mental health reasons. This may reflect the lack of access to primary care physicians in rural regions, with ratios of physicians per capita being between 30 to 50% greater in major urban centres compared to other regions [40], a situation that has barely evolved over the past 20 years [49]. General practitioners in rural areas may be especially overwhelmed and not able/willing to treat severe mental illnesses [50], preferring to refer to psychiatric care [51, 52]. Community psychosocial services are also lacking outside urban areas [53], which may result in a greater involvement of medical professionnals. For example, a study of Québec general practitioners’ practices in mental healthcare found that general practitioners of rural areas were 1.5 times more likely to refer to outpatient psychiatric clinics than general practitioners in urban or semi-urban areas, while they were less susceptible to refer to psychosocial services, psychologists offices, community organisms or crisis centres [53]. Another study in the United States supports the hypothesis that rural populations are less likely to rely on psychosocial services, with data suggesting that they are half as likely to initiate psychotherapy when needed as urban populations – but that they engaged in similar intensity and for similar lengths once initiated [54]. It is also possible that lack of access to first line mental health services outside of major urban centres, including primary care and non-medical resources [54], result in increased use of the emergency departments [55], thus increasing the odds of being evaluated by a psychiatrist. This would explain the findings that living in a rural zone influence the odds of reaching specialist care, but not the volume of specialist care used. Finally, it is also possible that existing resources are better known in smaller communities, and that fewer service points results in a more patient-centred, better coordinated care.

When adjusting for urbanization of the area of residence, closer proximity to hospital centres offering services upon referral also increased the odds of reaching specialized mental health care, but proximity to outreach services decreased the volume of psychiatric visits and improved continuity of psychiatric care. This may reflect a greater connection with community-based organisations that offer non-medical mental health services, or interaction with multidisciplinary teams such as Assertive Community Treatment where service users may see non-medical team members more often than the physician associated with the team. Given that nearly half of people with a NCRMD verdict had a history of criminal justice involvement, there is an opportunity to implement Forensic Assertive Community Treatment teams for people with a severe mental illness and who are believed to be at risk of committing an offense to address both their mental health needs and their criminogenic needs [56, 57]. These Forensic Assertive Community Treatment teams may also be offered to people with severe mental illness who are not justice-involved but who are considered at risk of violence or criminal justice involvement.

Another important trend that emerged from the findings was that living with a partner or family decreased the odds of accessing and receiving mental healthcare. While Andersen’s model of behavioral access to care purports that social support is a facilitator for health services use [48], it was not the case here. Instead, living with relatives decreased the odds of seeking mental healthcare, of reaching psychiatric care, and the volume of psychiatric visits. The empirical literature on social networks and mental health service use among people with severe mental illness tends to show that smaller social networks or lower social/family support are associated with greater inpatient service use [47, 58]. Epidemiological catchement area studies (in Montréal, Québec [59] and Baltimore, USA [60] respectively) have also shown that greater social support reduced the likelihood of reaching psychiatric care [59, 60]. It is possible that living with loved ones increases the self-perceived stigma both from the service user perspective and from the relatives, as the “marked difference that [is] negatively appraised” may become more apparent when cohabiting [61]. This may increase the preference for self-reliance, as stigma related to severe mental illness, especially when combined with that of a history of criminal justice involvement [9, 62, 63], may interfere with the ability and willingness of service users and their loved ones to fully participate in care [64]. Living with relatives may also increase the perceived ability to self-manage [65], as some may take the role of caregiver and provide some aspects of care, thus reducing the reliance on psychiatric visits or or delaying the referral to a psychiatrist by a family physician. This may be especially true in health systems that rely heavily on families and loved ones for daily care and help seeking. Another possible explanation is that families may protect their loved ones with mental illness from services that they do not find acceptable or that are accessed through judicial levers that families are not willing to use (e.g., involuntary treatment orders, involuntary admission) [66, 67]. Issues related to the acceptability of mental health services are important barriers to access and use of healthcare in Québec [68] and elsewhere [27], especially in a context where living with a severe mental illness is still heavily stigmatized. A narrative synthesis of access to services for another highly stigmatized group (people who inject drugs) has highlighted the strong role of non-judgmental health workers, high confidentiality, and flexibility of services in making services more acceptable to service users and increasing access [69]. In the context of mental health services, families may be unwilling to use levers to access care that may jeopardize their relationship with their loved one [66], that may subject them to discrimination or judgement from health workers, or that they may perceive themselves as unsafe or ineffective [27]. A fragmented system of care [70] may have also left many families and service users – especially those with concurrent substance use disorders – frustrated, having experienced considerable discrimination and disregard from some practitioners [71], and having received very little support in return. For some service users, the association may be reverse: people who are unable to access appropriate mental healthcare in a timely manner may be more susceptible to moving in with relatives and loved ones, such as parents, siblings or adult children. Regardless of the direction of the association, this finding is important as people who are found NCRMD are especially likely to victimize family members [72]. Resources should be invested to provide psycho-education and support to relatives [73], who are primary sources of financial, psychological and social support. This requires sufficient resources so that healthcare providers have the time to listen to families and engage with them, and that they are well-trained in the ethical and legal considerations that such a close collaboration with the family entails [74]. Relatives are too often in untenable positions, having to juggle seeking care, understanding the administrative maze and civil court provisions, and their own health and safety.

Finally, findings suggest that general practitioners, to the extent that they have a sustained relationship with their patient, may play an important role in the first line of identification of mental health problems. Nearly all participants who had a significant connection to a general practitioner were identified as having mental health needs and received primary mental health care in response to those needs. Yet, less than one in five participants were considered as significantly attached to a general practitioner, which is slightly lower than what is observed in the general population of the province of Québec during the same period [44], despite considerably higher needs. The decision to seek help for a health problem has been found to be associated to the sense of affinity to a primary care practice and the sense of trust in a primary care provider, especially for those who are socioeconomically disadvantaged [30]. Health policies should continue focusing on providing access to family physicians, supporting greater involvement from primary care clinicians, and access as appropriate to specialists in mental health, substance use and the plight of socially marginalized persons in prevention of criminal justice involvement. Those changes may require more structural transformations, such as to the mode of remuneration of physicians. The traditional fee-for-service adopted throughout Canada has shown to have deleterious effects on access to services by service users who are perceived as difficult or vulnerable, and thus less “cost-effective” [75]. Other models may be more appropriate for this population, especially as mental health care should be interprofessional [75]. For example, a study in Ontario robustly showed that a blended capitation model for primary care physicians was associated with better outcomes in terms of psychiatric health than a blended fee-for-service, as it may promote continuity of care and accessibility to services as well as promote interdisciplinary work [76].

### Limitations and future research

The present paper has some limitations. First, the use of governmental administrative health data limits us to linked medical services data, and does not provide insight into the use of other psycho-social services that can play a key role in risk assessment and management (e.g., psychological, counselling services, or spiritual counsellors for some). The analyses may also be conservative as some contacts with health services may not have been recognized as related to mental health reasons. We were also unable to adjust for the level of need associated to the mental illness, as any proxy we could have identified based on the governemental administrative database would have simultaneously been an indicator of access. Second, contextual factors are static and retrospective rather than dynamic and prospective, which limits the ability to draw causal inferences. The only address available to identify contextual factors were those at the time of the index offense (i.e., at the end of the observation period), which is true also for other variables such as living with relatives. It is thus possible that needs for mental health services influenced where the subject chose to reside and with whom. Finally, the data dates back to 2000–2005—however, relatively few changes have been made to the organization of MH services since that time, except for psychotherapy coverage by public insurance, to which access remains very difficult.

We would suggest that a replication study be done with a more recent sample. This study could take the form of a case-control study with a matched sample of service users who have not committed an NCRMD offense to identify difference in access and service use. While the present study relies entirely on administrative data, preventing the examination of psychological factors, such as beliefs and attitudes, that may underlay decisions to seek and receive different types of services [30], this case-control study could adopt a mixed design to shed light on “why” and “how” services are accessed and received. This could allow us to understand whether geographic inequity in access to care is similar among forensic and nonforensic samples, and identify practices and service trajectories that may play a role in violence prevention, all else being equal.

## Conclusion

﻿ The findings reveal inequities in opportunities to access mental health services in the trajectories of forensic service users. Geographic factors and the presence of relatives in the lives of service users have a greater influence on the odds of seeking, reaching and receiving mental healthcare than factors related to the intensity of needs. Engagement with family members – both in the clinical setting with family-centred services and in the research setting with participatory action research – is an area that is vastly underprioritized, especially as close relatives are the most likely victims of NCRMD offenses. Family-centred services are potentially an important lever to improve engagement with mental healthcare among people who have a severe mental illness and who have behaviors that make them at risk of criminal justice involvement. Access to mental health services may prevent violence and criminal justice involvement for a subset of mental health service users, as long as services are appropriate in nature and intensity.

## Supplementary Information

**Additional file 1: Table S1.** Characteristics and index offenses of participants included and excluded from the present analyses.

## Data Availability

The administrative health data that support the findings of this study are available from the *Régie d’assurance maladie du Québec* and *MED-ECHO* but restrictions apply to the availability of these data, which were used under license for the current study, and so are not publicly available. Other data from the NTP study analysed during the current study are not publicly available due to their highly sensitive content and imperative to protect the anonymity of participants. All questions regarding the availability of the data should be directed to the corresponding author.

## Footnotes

1 In Canada, Review Boards are independent administrative tribunals ﻿responsible for rendering decisions regarding persons found NCRMD.
