# Supplementary material for: Barriers and facilitators of access and utilization of mental health services among forensic service users along the care pathway
Source: BMC Health Serv Res. 2022 Dec 7;22:1495. doi: 10.1186/s12913-022-08848-9 (PMC9730649; doi:10.1186/s12913-022-08848-9)
Supplement: Supplementary file 1 — Additional file 1: Table S1. Characteristics and index offenses of participants included and excluded from the present analyses. [file 12913_2022_8848_MOESM1_ESM.docx]

**Online supplement**

Table S1

*Characteristics and index offenses of participants included and excluded from the present analyses*

| Sociodemographic and clinical characteristics | | | Offense characteristics and criminal justice history | | |
| --- | --- | --- | --- | --- | --- |
| Variables | **Included  (n=753)** | **Excluded  (n=341)** |  | **Included  (n=753)** | **Excluded  (n=341)** |
| Gender, % |  |  | Most severe index offense, % |  |  |
| Women | 14.5 | 16.6 | Causing death or attempting to cause death | 4.9 | 3.8 |
| Men | 85.5 | 83.4 | Sexual offense | 1.5 | 2.2 |
| Age, *M* (*SD*) | 36.7 (12.4) | 35.7 (12.9) | 1st degree assault | 5.2 | 6.8 |
| Civil status, % |  |  | Other assault | 19.8 | 19.6 |
| In a relationship | 15.7 | 12.7 | Threats | 22.0 | 18.3 |
| Single | 84.3 | 87.3 | Other against the person | 8.2 | 13.0 |
| Born in Canada, % | 62.7 | 64.5 | Property offenses | 19.9 | 21.1 |
| Indigenous, % | 1.0 | 1.8 | Administration of justice | 4.9 | 3.8 |
| Revenue, % |  |  | Other | 13.7 | 11.4 |
| Own paid work (or partner’s) | 16.7 | 13.4 | Criminal history, % | 45.0 | 48.7 |
| Pensions, welfare, disability | 74.8 | 72.4 | NCRMD history, % | 8.5 | 12.6 |
| Other | 8.5 | 14.2 |  |  |  |
| Primary diagnosis, % |  |  |  |  |  |
| Psychotic disorder | 64.2 | 69.6 |  |  |  |
| Mood disorder | 29.6 | 25.1 |  |  |  |
| Other | 6.2 | 5.3 |  |  |  |
| Concurrent personality disorder, % | 9.7 | 11.3 |  |  |  |
| Concurrent substance use disorder, % | 30.0 | 26.1 |  |  |  |

*Note*. Weighted statistics.
